# Efficacy of Lumateperone in depression associated with bipolar II disorder: a pooled analysis of late-phase clinical trials

**DOI:** 10.1017/S1092852925100564

**Published:** 2025-09-29

**Authors:** Suresh Durgam, Hassan Lakkis, Susan G. Kozauer, Changzheng Chen, Roger S. McIntyre

**Affiliations:** 1 Intra-Cellular Therapies, a Johnson & Johnson Company, Bedminster, NJ, USA; 2 Department of Psychiatry, University of Toronto, Toronto, ON, Canada; 3 Department of Pharmacology and Toxicology, University of Toronto, Toronto, ON, Canada

**Keywords:** Adjunctive therapy, Antipsychotic agents, bipolar depression, bipolar II disorder, lumateperone

## Abstract

**Objective:**

Treatment options are limited for depressive episodes in patients with bipolar II disorder. This post hoc analysis evaluated the efficacy of lumateperone in three pooled short-term, Phase 3 studies in patients with a major depressive episode (MDE) associated with bipolar II disorder.

**Methods:**

This post hoc analysis pooled data from patients (18–75 years) with *DSM-5* diagnosed bipolar II disorder experiencing an MDE in randomized, double-blind, placebo-controlled studies of lumateperone 42 mg monotherapy (Study 401, Study 404) and adjunctive therapy to lithium or valproate (Study 402). Primary and key secondary outcomes were change from baseline in Montgomery-Åsberg Depression Rating Scale (MADRS) Total and Clinical Global Impression Scale-Bipolar Version-Severity (CGI-BP-S) scores. Safety was also assessed.

**Results:**

Lumateperone significantly improved MADRS Total score at Day 43 in the bipolar II population (placebo, *n* = 87; lumateperone, *n* = 87; least squares mean difference vs. placebo [LSMD], −4.0; *P* < .05). In the bipolar II population, lumateperone significantly improved CGI-BP-S Total (LSMD, −1.0; *P* < .05), Depression (LSMD, −0.5; *P* < .05), and Overall Bipolar Illness scores (LSMD, −0.5; *P* < .05) compared with placebo at Day 43. No new safety signals were identified, with minimal risk of extrapyramidal symptoms, cardiometabolic abnormalities, or prolactin elevation.

**Conclusions:**

Lumateperone 42 mg monotherapy or adjunctive therapy significantly improved symptoms of depression and disease severity in patients with bipolar II disorder across Phase 3 studies. Lumateperone was generally well tolerated. These results support lumateperone 42 mg to treat MDEs associated with bipolar II disorder.

## Introduction

Bipolar II disorder is a chronic mental illness characterized by recurring cycles of manic/hypomanic and depressive episodes.^1^ In the United States, bipolar II disorder has a lifetime prevalence of 1.1%.^2^ Bipolar II disorder has unique characteristics from bipolar I disorder, such as a greater frequency among women than men,^3^ an increased incidence of rapid cycling and comorbidities,^4^ and an increased likelihood to report childhood trauma compared with bipolar I disorder.^5^ As depressive episodes are usually the first to clinically manifest, bipolar II disorder is often misdiagnosed as unipolar depression, which can delay the time to accurate diagnosis.^6^

By definition, hypomania is less severe than mania, leading to the perception that bipolar II disorder is less burdensome than bipolar I disorder.^7^ ^,^^8^ However, depressive episodes are approximately 30 times more prevalent than hypomanic episodes in bipolar II disorder^9^ and are associated with functional impairment and decreased quality of life.^8^ ^,^^10^ There is ample evidence that a non-negligible percentage of patients who meet the criteria for treatment-resistant depression have bipolar II disorder as their principal diagnosis.^11^ Functional impairment associated with bipolar II disorder is comparable to that of bipolar I disorder,^7^ which highlights the burden of bipolar II disorder. In addition, recent evidence suggests that the suicide rate is similar or even greater in patients with bipolar II disorder compared with bipolar I disorder.^1^ ^,^^7^ ^,^^12^

Bipolar II disorder has been understudied, and there are fewer published data for the bipolar II population compared with the bipolar I population.^7^ The trials that investigate bipolar II disorder are often underpowered or fail to report the results for the bipolar II population separately from bipolar I, limiting the data that can be analyzed for this group of patients.^7^ Despite demonstrating efficacy for treating bipolar depression, antipsychotics are associated with various undesirable side effects that include cardiometabolic abnormalities, weight gain, extrapyramidal symptoms (EPS), and hyperprolactinemia.^3^ ^,^^13^ ^,^^14^ Adverse effects are associated with nonadherence rates of 34% to 80% in patients with bipolar disorder and have been shown to impair functionality and decrease quality of life in patients with schizophrenia.^15^ ^,^^16^ Antidepressants have also been used to treat bipolar II depression, but do not have established or proven efficacy.^17^ Pharmacotherapies that are effective at treating episodes of depression in bipolar II disorder and have favorable safety profiles that diminish the impact of adverse effects on adherence, quality of life, and functionality are needed.

Medication options are limited for depression in bipolar II disorder, for which only quetiapine (including extended-release quetiapine)^18^ ^,^^19^ and lumateperone^20^ are US Food and Drug Administration (FDA) approved.^3^ Lumateperone is a mechanistically novel antipsychotic that is FDA approved for the treatment of schizophrenia and depressive episodes associated with bipolar I or bipolar II disorder as monotherapy and as adjunctive therapy with lithium or valproate.^20^ ^–^^22^ Lumateperone is a potent serotonin 5-HT_2A_ receptor antagonist, a dopamine D_2_ receptor presynaptic partial agonist and postsynaptic antagonist, a D_1_ receptor-dependent indirect modulator of glutamatergic α-amino-3-hydroxy-5-methyl-4-isoxazolepropionic acid (AMPA) and N-methyl-d-aspartate (NMDA) currents, and a serotonin reuptake inhibitor.^22^ ^–^^24^

The efficacy and safety of lumateperone were investigated in three Phase 3, short-term, randomized, double-blind, placebo-controlled studies in patients with a major depressive episode (MDE) associated with bipolar I or bipolar II disorder.^25^ ^–^^27^ In two studies (NCT03249376 for monotherapy and NCT02600507 for adjunctive therapy, also known as Studies 404 and 402, respectively), lumateperone treatment for 6 weeks significantly improved symptoms of depression compared with placebo, as measured by the primary endpoint, change from baseline in Montgomery-Åsberg Depression Rating Scale (MADRS) Total score.^26^ In another monotherapy trial (NCT02600494; Study 401), numerical improvements in MADRS Total score with 6-week lumateperone therapy were seen; however, a statistically significant difference from placebo was not observed, likely due to a high placebo response.^27^ In all three studies, 6-week lumateperone monotherapy and lumateperone adjunctive therapy with lithium or valproate were generally well tolerated in patients with bipolar depression, with minimal to no changes in metabolic parameters, weight gain, prolactin, and vital signs, and low risk of EPS.^25^ ^–^^27^

These Phase 3 studies were not powered to detect treatment differences in the subgroups of bipolar I or bipolar II disorder. In order to assess the efficacy and safety of lumateperone in patients with bipolar II disorder, we conducted a post hoc analysis of the pooled efficacy and safety data from patients with bipolar II disorder experiencing an MDE who were treated with lumateperone 42 mg monotherapy or adjunctive therapy across these three studies.

## Methods

### Study designs and patient population

Data from a subpopulation of patients with bipolar II disorder were pooled from three similarly designed, randomized, double-blind, placebo-controlled trials of lumateperone in patients with bipolar I or bipolar II disorder experiencing an MDE (NCT03249376, NCT02600507, NCT02600494).^25^ ^–^^27^ Study design details for these studies have been previously described.^25^ ^–^^27^ All three studies included a 6-week treatment period with lumateperone as monotherapy or adjunctive therapy followed by 2 weeks of safety follow-up. Each study included patients 18 to 75 years old with a clinical diagnosis of bipolar I or bipolar II disorder according to *Diagnostic and Statistical Manual of Mental Disorders* (Fifth Edition) criteria. Patients were required to have depression of at least moderate severity as measured by a MADRS^28^ Total score ≥20 and Clinical Global Impression Scale-Bipolar Version-Severity (CGI-BP-S)^29^ Depression and Overall Bipolar Illness subscores of ≥4, and have a Young Mania Rating Scale (YMRS)^30^ Total score ≤12 at screening and baseline. Patients were experiencing an MDE for ≥2 weeks but ≤6 months prior to screening, with symptoms causing clinically significant distress or impaired function. In Study 402, patients must also have had treatment with either lithium or valproate for ≥28 days and had a history of inadequate therapeutic response of depressive symptoms.

Patients were stratified by bipolar I or bipolar II diagnosis in all three studies before being randomized to placebo or lumateperone as monotherapy in Studies 404 and 401 or as adjunctive therapy to lithium or valproate in Study 402. Lumateperone 42 mg (the FDA-approved recommended dose and focus of this analysis) or placebo was administered to patients once daily in the evening for 6 weeks. Safety and efficacy assessments were conducted at weekly clinic visits on Days 8, 15, 22, 29, 36, and 43, and at a final follow-up visit approximately 2 weeks following the last dose of study medication. All studies were approved by the appropriate Institutional Review Boards/Independent Ethics Committee, and written informed consent was obtained from each patient before entering the study. The authors assert that all procedures contributing to this work comply with the ethical standards of the relevant national and institutional committees on human experimentation and with the Helsinki Declaration of 1975, as revised in 2008.

### Outcome measures

In all three studies, the primary efficacy endpoint was the change from baseline to Day 43 in MADRS Total score. The CGI-BP-S scale (Total score and subscale scores) was used to assess changes in disease severity from baseline to Day 43; the subscale scores were CGI-BP-S Depression, CGI-BP-S Mania, and CGI-BP-S Overall Bipolar Illness, the latter reflecting the clinician’s global impression of the patient’s current state of bipolar illness. Safety assessments included adverse events (AEs), clinical laboratory evaluations, electrocardiograms, physical and neurological examinations, and vital sign measurements. EPS were assessed by the Simpson-Angus Scale (SAS),^31^ Barnes Akathisia Rating Scale (BARS),^32^ and Abnormal Involuntary Movement Scale (AIMS).^33^ Mania was monitored using the YMRS,^30^ and suicidality was evaluated with AEs and the Columbia Suicide Severity Rating Scale (C-SSRS).^34^

Additional outcomes included quality of life, which was assessed using the Quality of Life Enjoyment and Satisfaction Questionnaire-Short Form (Q-LES-Q-SF) percent score.

### Post hoc analyses

For all analyses, the subgroup of patients with bipolar II disorder at baseline was pooled for the three studies. MADRS Total and CGI-BP-S Total and subscale scores were evaluated using a mixed-effects model for repeated measures (MMRM) in the intent-to-treat (ITT) population, defined as all patients who received ≥1 dose of study medication and had a valid baseline and ≥1 valid postbaseline MADRS assessment. The MMRM model included visit, treatment group, site (pooled site), adjunctive therapy type (for Studies 401 and 404: monotherapy; for Study 402: lithium or valproate), bipolar disorder stratification at baseline (for ITT analysis: bipolar I or bipolar II disorder; for bipolar II subgroup analysis: all bipolar II disorder) as factors, baseline score as a covariate, and interaction terms for treatment group-by-visit and visit-by-baseline score. An unstructured covariance matrix was used to model the covariance within patient scores. Q-LES-Q-SF percent score was measured using analysis of covariance—last observation carried forward (ANCOVA-LOCF). Treatment-emergent AEs (TEAEs) were summarized using descriptive statistics by treatment group in the safety population, defined as patients receiving ≥1 dose of study drug. Clinician-rated EPS scale (AIMS, BARS, SAS) Total scores, and YMRS Total score were analyzed via ANCOVA-LOCF.

## Results

### Patient population

**Figure 1.**
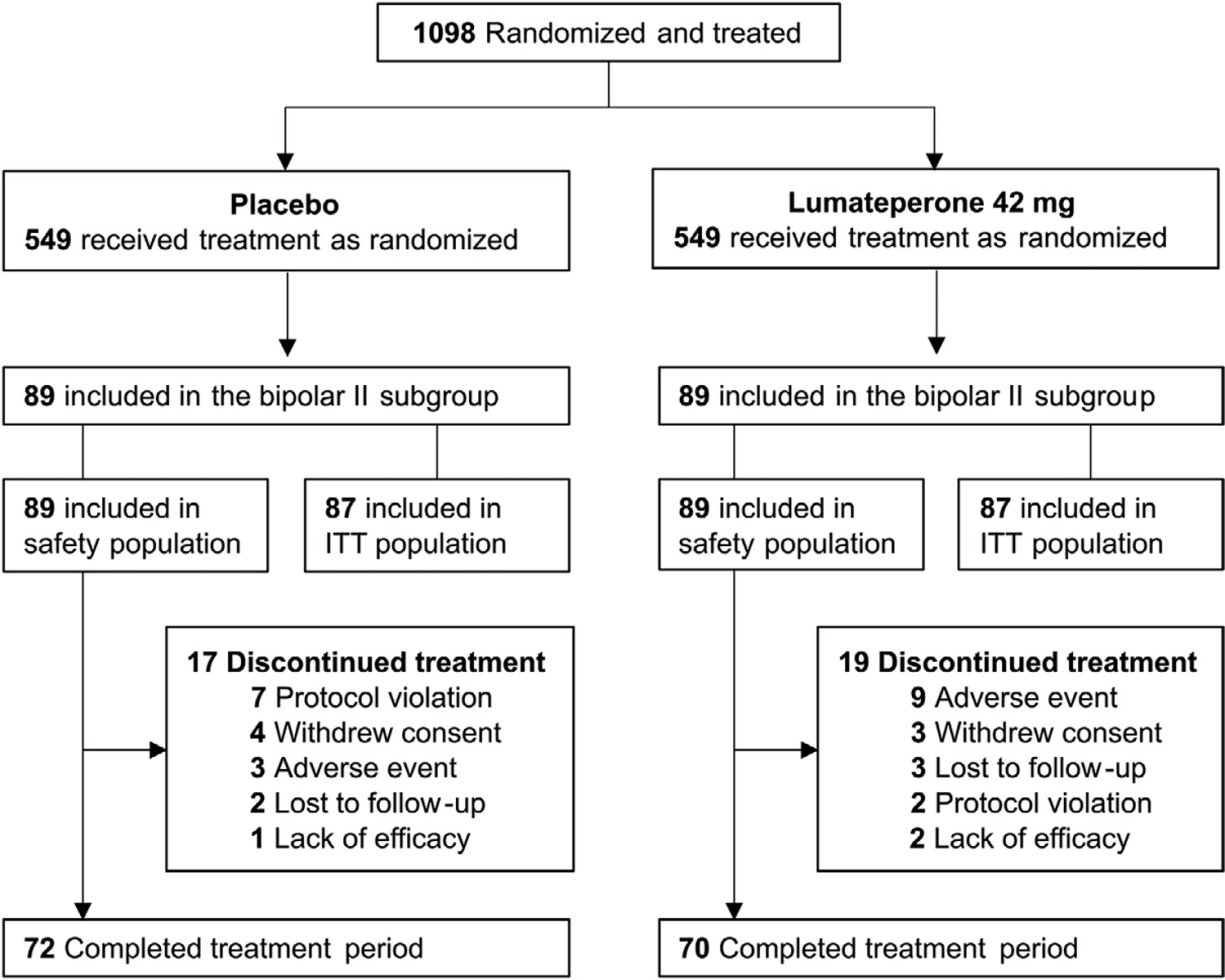
Patient disposition. ITT, intent-to-treat.

A total of 1098 patients with bipolar I or bipolar II disorder (placebo, *n* = 549; lumateperone 42 mg, *n* = 549) were included in the pooled safety population for this analysis, with 178 patients (16.2%) diagnosed with bipolar II disorder (placebo, *n* = 89; lumateperone, *n* = 89) (Figure 1). The pooled ITT population from all studies comprised 1067 patients with bipolar I or bipolar II disorder (placebo, *n* = 539; lumateperone 42 mg, *n* = 528). Of these, 174 patients (16.3%) were diagnosed with bipolar II disorder (placebo, *n* = 87; lumateperone, *n* = 87) (Figure 1). In patients with bipolar II disorder, the most common cause of discontinuation from treatment was AEs in the lumateperone group and protocol violation in the placebo group (Figure 1).

At baseline, demographics and disease characteristics were similar between the lumateperone and placebo groups (Table 1). The majority of participants were White (placebo, 88.8%; lumateperone, 85.4%) and women (placebo, 56.2%; lumateperone, 53.9%) with a mean age of 41 to 42 years old. Mean baseline MADRS Total score (placebo, 31.5; lumateperone, 30.9) and CGI-BP-S depression subscore (4.5 for both groups) indicate moderate to severe depression at baseline (Table 1). The mean age at first bipolar II diagnosis was 32 years (range, 8–70 years). Comparing the three studies pooled for this analysis, patients were mostly female, White, in their late 30s to early 40s (mean age: 37.5–44.9 years old), and had first been diagnosed with bipolar disorder in their early-to-mid 30s (mean age: 30.2–35.8 years). Additionally, patients all had moderate to severe depression symptoms at baseline, based on mean MADRS Total score (28.8–36.7).

**Table 1.** Baseline Demographics and Clinical Characteristics for Pooled Bipolar II Population (Safety Population)

	Placebo	Lumateperone 42 mg
	(*n* = 89)	(*n* = 89)
Age, mean (range), years	40.8 (19–74)	41.4 (19–71)
Sex, %		
Women	53.9	56.2
Men	46.1	43.8
Race, %^a^		
White	85.4	88.8
Black	10.1	7.9
Other	4.5	3.4
Hispanic or Latino ethnicity, %	10.1	6.7
Age at first bipolar disorder diagnosis, mean (range), years	32.1 (11–61)	32.1 (8–70)
Number of lifetime depressive episodes, %		
1–9	80.2	76.7
10–20	16.3	22.1
>20	3.5	1.2
MADRS Total score, mean (SD)	30.9 (5.55)	31.5 (6.10)
CGI-BP-S, mean (SD)		
Total score	10.0 (1.34)	10.1 (1.39)
Depression subscore	4.5 (0.59)	4.5 (0.64)
Overall Bipolar Illness subscore	4.4 (0.58)	4.4 (0.62)
Mania subscore	1.1 (0.42)	1.2 (0.40)
Q-LES-Q-SF percent score, mean (SD)	38.4 (12.88)	35.8 (13.84)

Abbreviations: CGI-BP-S, Clinical Global Impression Scale-Bipolar Version-Severity; MADRS, Montgomery-Åsberg Depression Rating Scale; Q-LES-Q-SF, Quality of Life Enjoyment and Satisfaction Questionnaire-Short Form.

a Due to rounding, percentages may not add to 100.

### Efficacy

**Figure 2.**
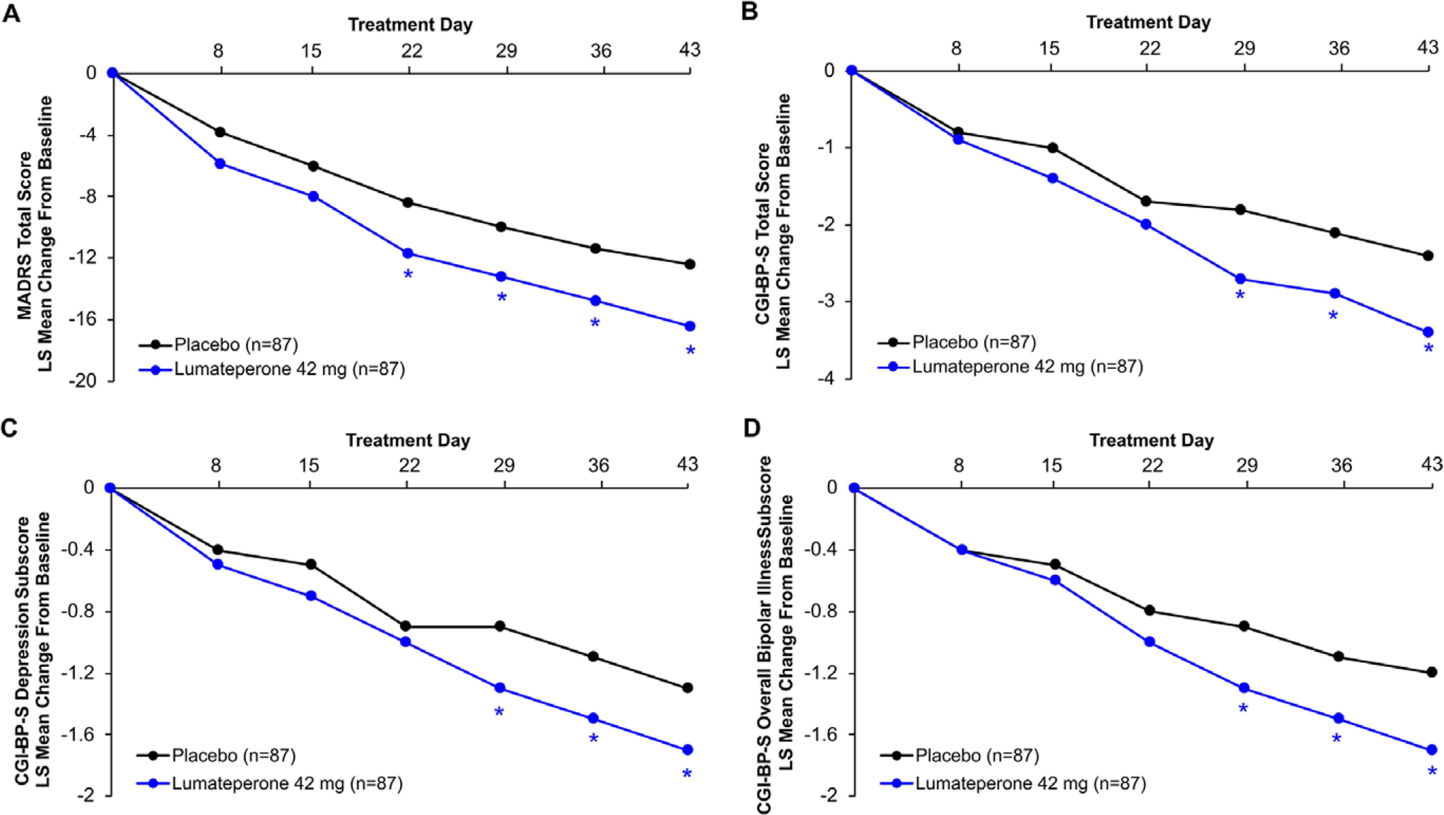
Mean change from baseline in efficacy parameters in pooled bipolar II population (ITT). (A) MADRS Total score; (B) CGI-BP-S Total score; (C) CGI-BP-S Depression subscore; (D) CGI-BP-S Overall Bipolar Illness subscore. *P < .05. LSMD vs. placebo. MMRM. CGI-BP-S, Clinical Global Impression Scale-Bipolar Version-Severity; ITT, intent-to-treat; LS, least squares; LSMD, least squares mean difference; MADRS, Montgomery-Åsberg Depression Rating Scale; MMRM, mixed-effects model for repeated measures.

Lumateperone significantly improved MADRS Total score at Day 43 in the pooled bipolar II population compared with placebo (least squares [LS] mean change from baseline: placebo, −12.4, lumateperone, −16.4; least squares mean difference vs. placebo [LSMD], −4.0; 95% CI, −7.2 to −0.7; effect size, −0.39; *P* < .05) (Figure 2A and Table 2). Significant improvements with lumateperone vs. placebo were seen beginning at Day 22, with continuing significant improvement throughout the subsequent visits (Figure 2A). Similarly, lumateperone treatment in the pooled overall bipolar I and bipolar II population was associated with significant improvement in the primary endpoint, MADRS Total score change from baseline to Day 43, compared with placebo (LS mean change: placebo, −14.8, lumateperone, −17.3; LSMD, −2.5; 95% CI, −3.8 to −1.2; *P* < .001) (Supplementary Figure S1).

**Table 2.** Change in Efficacy Parameters at Day 43 in Pooled Bipolar II Population (ITT)

	Placebo		Lumateperone 42 mg					
	(*n* = 87)		(*n* = 87)		Comparison with placebo			
	LS mean change	SE	LS mean change	SE	LSMD	95% CI	Effect size	*P* value
MADRS Total score	−12.4	1.17	−16.4	1.19	−4.0	−7.2, −0.7	−0.39	0.016
CGI-BP-S Total score	−2.4	0.29	−3.4	0.30	−1.0	−1.8, −0.2	−0.39	0.016
CGI-BP-S Depression subscore	−1.3	0.14	−1.7	0.15	−0.5	−0.9, −0.1	−0.37	0.021
CGI-BP-S Overall Bipolar Illness subscore	−1.2	0.14	−1.7	0.14	−0.5	−0.9, −0.1	−0.40	0.013
CGI-BP-S Mania subscore	0.0	0.06	0.0	0.06	0.0	−0.2, 0.1	−0.08	0.614
Q-LES-Q-SF percent score^a^	10.9	1.88	17.2	1.91	6.3	1.1, 11.4	0.38	0.017

Abbreviations: CGI-BP-S, Clinical Global Impression Scale-Bipolar Version-Severity; ITT, intent-to-treat; LS, least squares; LSMD, least squares mean difference; MADRS, Montgomery-Åsberg Depression Rating Scale; Q-LES-Q-SF, Quality of Life Enjoyment and Satisfaction Questionnaire-Short Form.

a An increase in Q-LES-Q-SF percent score indicates improvement.

In the pooled bipolar II population, lumateperone significantly improved disease severity at Day 43 compared with placebo according to CGI-BP-S Total score (LS mean change: placebo, −2.4, lumateperone, −3.4; LSMD, −1.0; 95% CI, −1.8 to −0.2; effect size, −0.39; *P* < .05), CGI-BP-S Depression subscore (LS mean change: placebo, −1.3, lumateperone, −1.7; LSMD, −0.5; 95% CI, −0.9 to −0.1; effect size, −0.37; *P* < .05), and CGI-BP-S Overall Bipolar Illness subscore (LS mean change: placebo, −1.2, lumateperone, −1.7; LSMD, −0.5; 95% CI, −0.9 to −0.1; effect size, −0.40; *P* < .05) (Figure 2B–D and Table 2).

### Safety

In the pooled bipolar II safety population, the rate of TEAEs was slightly higher in the lumateperone group (65.2%) compared with placebo (48.3%) (Table 3). Drug-related TEAEs occurred in 30.3% of the placebo group and 55.1% of the lumateperone group (Table 3). TEAEs that occurred in ≥5% of patients and more than twice the rate of placebo were headache (placebo, 6.7%; lumateperone, 18.0%), dizziness (placebo, 5.6%; lumateperone, 12.4%), somnolence (placebo, 2.2%; lumateperone, 15.7%), postural dizziness (placebo, 1.1%; lumateperone, 10.1%), and nausea (placebo, 3.4%; lumateperone, 10.1%) (Table 3). The majority of TEAEs were mild to moderate in severity, with one patient (1.1%) in the placebo group and two patients (2.2%) in the lumateperone group experiencing severe TEAEs. AEs led to discontinuation of treatment in three patients (3.4%) in the placebo group and nine patients (10.1%) in the lumateperone group (Table 3). There was only one serious TEAE of aggression in the placebo group, which led to discontinuation. No deaths occurred in the bipolar II population across the studies (Table 3).

**Table 3.** Adverse Events in Pooled Bipolar II Population (Safety Population)

	Placebo	Lumateperone 42 mg
	(*n* = 89)	(*n* = 89)
	*n* (%)	*n* (%)
Number of patients with		
≥1 TEAE	43 (48.3)	58 (65.2)
Drug-related TEAE	27 (30.3)	49 (55.1)
SAE	1 (1.1)	0
Discontinued treatment due to AE	3 (3.4)	9 (10.1)
Patients who died	0	0
TEAEs occurring ≥5% and more than twice the rate of placebo (by preferred term)		
Headache	6 (6.7)	16 (18.0)
Somnolence	2 (2.2)	14 (15.7)
Dizziness	5 (5.6)	11 (12.4)
Postural dizziness	1 (1.1)	9 (10.1)
Nausea	3 (3.4)	9 (10.1)

Abbreviations: AE, adverse event; SAE, serious adverse event; TEAE, treatment-emergent adverse event.

There was one TEAE of mania in a patient with bipolar II disorder treated with lumateperone and no TEAEs of hypomania in either treatment group. The LS mean change from baseline to end of treatment in YMRS Total score was small (placebo, −0.8; lumateperone, −0.6), indicating no worsening of mania in the pooled bipolar II population. Similarly, change from baseline to Day 43 in the CGI-BP-S Mania subscore (in the ITT population) with lumateperone was minimal and similar to that of placebo (LS mean change, 0.0; LSMD, 0.0; 95% CI, −0.2 to 0.1; *P* = .61) (Table 2). Emergence of suicidal ideation based on the C-SSRS occurred at a lower rate in the lumateperone group (4.1%) compared with the placebo group (8.0%). No patients with bipolar II disorder across all studies reported suicidal behavior.

According to a narrow standard Medical Dictionary for Regulatory Activities (MedDRA) query, no EPS-related TEAEs occurred in either the placebo or lumateperone groups in the pooled bipolar II population. No statistically significant changes from baseline to end of treatment occurred in the BARS, AIMS, or SAS scales in either treatment group (*P* > .05) (Supplementary Table S1).

Changes in weight and body morphology were minimal and similar between the placebo and lumateperone groups in the pooled bipolar II population (Supplementary Table S2). No patients with bipolar II disorder who received lumateperone experienced clinically significant (PCS) weight increase (≥7% increase from baseline), and one patient (1.2%) in the placebo group experienced PCS weight increase (Supplementary Table S2). Similarly, no patients in the lumateperone group and one patient (1.2%) in the placebo group experienced PCS weight decrease (≥7% decrease from baseline) (Supplementary Table S2). There were no clinically significant differences in prolactin, liver enzymes, or cardiometabolic parameters, including total cholesterol, low-density lipoprotein cholesterol, high-density lipoprotein cholesterol, triglycerides, glucose, and insulin, between the placebo and lumateperone groups. Mean increases in prolactin levels were higher in the placebo group (mean change, 6.87) compared with the lumateperone group (mean change, 0.96) (Supplementary Table S2). No patients had a QTcF (Fridericia corrected QT) interval >480 ms.

### Quality of life

Lumateperone also significantly improved the Q-LES-Q-SF percent score at Day 43 compared with placebo (LS mean change: placebo, 10.9, lumateperone, 17.2; LSMD, 6.3; 95% CI, 1.1 to 11.4; effect size, 0.38; *P* < .05) (Table 2).

## Discussion

In this pooled analysis of three late-phase placebo-controlled studies, lumateperone monotherapy or adjunctive therapy significantly improved depressive symptoms compared with placebo in patients with bipolar II disorder. Lumateperone treatment was associated with statistically significant improvement in MADRS Total score starting at Day 22, with continual improvement until Day 43.

It is of note that while one of the three pooled studies, Study 401, did not meet its primary endpoint (likely due to high placebo response), this pooled analysis of three studies demonstrated statistical separation from placebo in the bipolar II disorder population. To achieve more robust estimates and detect significant effects based on a larger sample size, this analysis pooled patients on lumateperone monotherapy and adjunctive therapy, mirroring real-world practice that incorporates a variety of treatment strategies. Based on baseline demographics and disease characteristics, the patient populations in the three studies were homogeneous enough to pool the data without introducing a risk of type I error.

Most of the published studies investigating antipsychotics for bipolar disorder have only included bipolar I disorder in their populations, including studies of olanzapine-fluoxetine,^35^ lurasidone,^36^ ^,^^37^ and cariprazine.^38^ ^–^^40^ Efficacy results from this pooled analysis (MADRS Total mean change at Day 43 for lumateperone, −16.4) were similar to results from an analysis of quetiapine (the only other medication approved for bipolar II depression) pooled monotherapy data that showed a mean change in MADRS Total score of −15.58 from the bipolar subgroups of four randomized, bipolar II depression studies.^41^ A study of cariprazine investigated efficacy for depression in a combined bipolar I and bipolar II disorder population; however, the study did not meet its primary endpoint and, as such, the bipolar II disorder results were not presented.^42^

Efficacy of lumateperone in patients with bipolar II disorder was additionally supported by significant improvements in disease severity as indicated by CGI-BP-S Total score, CGI-BP-S Depression subscore, and CGI-BP-S Overall Bipolar Illness subscore. Lumateperone also significantly improved quality of life in the bipolar II population, as measured by the Q-LES-Q-SF. Impaired quality of life in bipolar II disorder is especially troublesome, with poorer health-related quality of life than in bipolar I disorder.^43^

Six-week treatment with lumateperone was generally well tolerated in patients with bipolar II disorder. The safety results are similar to monotherapy in bipolar depression with mixed features or major depressive disorder with mixed features,^44^ long-term open-label monotherapy in bipolar depression,^45^ and pooled safety analyses of lumateperone monotherapy in bipolar depression^46^ and schizophrenia,^47^ with no new safety signals detected. The majority of TEAEs were mild to moderate in severity, and AEs that occurred at rates of ≥5% and twice that of placebo were dizziness, headache, somnolence, and nausea. Rates of treatment-emergent hypomania and suicidal ideation were low. Minimal changes in the CGI-BP-S Mania subscore and YMRS Total score indicate no increase in mania in the bipolar II population during the studies. There was one TEAE of mania in a patient diagnosed with bipolar II disorder and treated with lumateperone. This TEAE may indicate initial misdiagnosis or a progression from bipolar II to bipolar I disorder, which may occur in a minority (5%–17%) of adult patients.^48^ ^,^^49^

Second-generation antipsychotics are associated with various side effects, including EPS, metabolic abnormalities, and hyperprolactinemia.^20^ No EPS-related TEAEs were reported in patients with bipolar II disorder who were in the lumateperone group. Weight and body morphology measurements were stable, and there were no significant changes in cardiometabolic parameters, including cholesterol, triglycerides, glucose, or insulin. Additionally, no meaningful changes in prolactin occurred with lumateperone.

Limitations of this study include the post hoc design, as analyses were not defined prior to the study. Additionally, the pooling of patients on monotherapy and adjunctive therapy in the analyses precludes comment on the efficacy of monotherapy or adjunctive therapy alone in the bipolar II population. None of the trials had active controls for comparison, and the generalizability of the findings may be limited due to the exclusion of patients with imminent suicidal risk, treatment-resistant illness, and serious comorbid medical or psychiatric illnesses.

## Conclusion

In this post hoc analysis investigating the pooled efficacy and safety of lumateperone in three short-term, randomized, placebo-controlled studies, lumateperone 42 mg significantly improved symptoms of depression and disease severity in people with bipolar II disorder. Treatment with lumateperone was also generally well tolerated in this population. These results support the efficacy and safety of lumateperone 42 mg for the treatment of MDEs associated with bipolar II disorder.

## Supporting information

Durgam et al. supplementary material Durgam et al. supplementary material

## Data Availability

Data will be made available on reasonable request, subject to review and meeting criteria. Registration Clinicaltrials.gov: NCT02600494, NCT03249376, NCT02600507.

## Supplementary material

The supplementary material for this article can be found at http://doi.org/10.1017/S1092852925100564.
