# Supplementary material for: Efficacy of Lumateperone in depression associated with bipolar II disorder: a pooled analysis of late-phase clinical trials
Source: CNS Spectr. 2025 Sep 29;30(1):e80. doi: 10.1017/S1092852925100564 (PMC13064759; doi:10.1017/S1092852925100564)
Supplement: Durgam et al. supplementary material [file S1092852925100564sup001.docx]

**Supplement for:**

**Efficacy of Lumateperone in Depression Associated With Bipolar II Disorder: A Pooled Analysis of Late-Phase Clinical Trials**

Suresh Durgam, MD^1^; Hassan Lakkis, PhD^1^; Susan G Kozauer, MD^1^; Changzheng Chen, PhD^1^; Roger S. McIntyre, MD^2^

^1^Intra-Cellular Therapies, a Johnson & Johnson Company, Bedminster, NJ, USA

^2^ Department of Psychiatry, University of Toronto, Toronto, ON, Canada

**Corresponding author name and contact information:**

Suresh Durgam, MD

Intra-Cellular Therapies, a Johnson & Johnson Company

135 US Highway 202/206, Suite 6

Bedminster, NJ 07921

Phone: +1-917-991-3134

Email: sdurgam@itci-inc.com

**Contents**

[Supplemental Figure S1. Mean Change From Baseline in MADRS Total Score in Overall Population with Bipolar I or Bipolar II Disorder (ITT) 2](#_Toc191625091)

[Supplemental Table S1. Mean Change From Baseline to EOT in Clinician-Rated Motor Scales in Pooled Bipolar II Population (Safety Population) 3](#_Toc191625092)

[Supplemental Table S2. Change in Body Morphology and Laboratory Parameters at EOT in Pooled Bipolar II Population (Safety Population) 4](#_Toc191625093)

# **Supplemental Figure S1. Mean Change From Baseline in MADRS Total Score in Overall Population with Bipolar I or Bipolar II Disorder (ITT)**

**
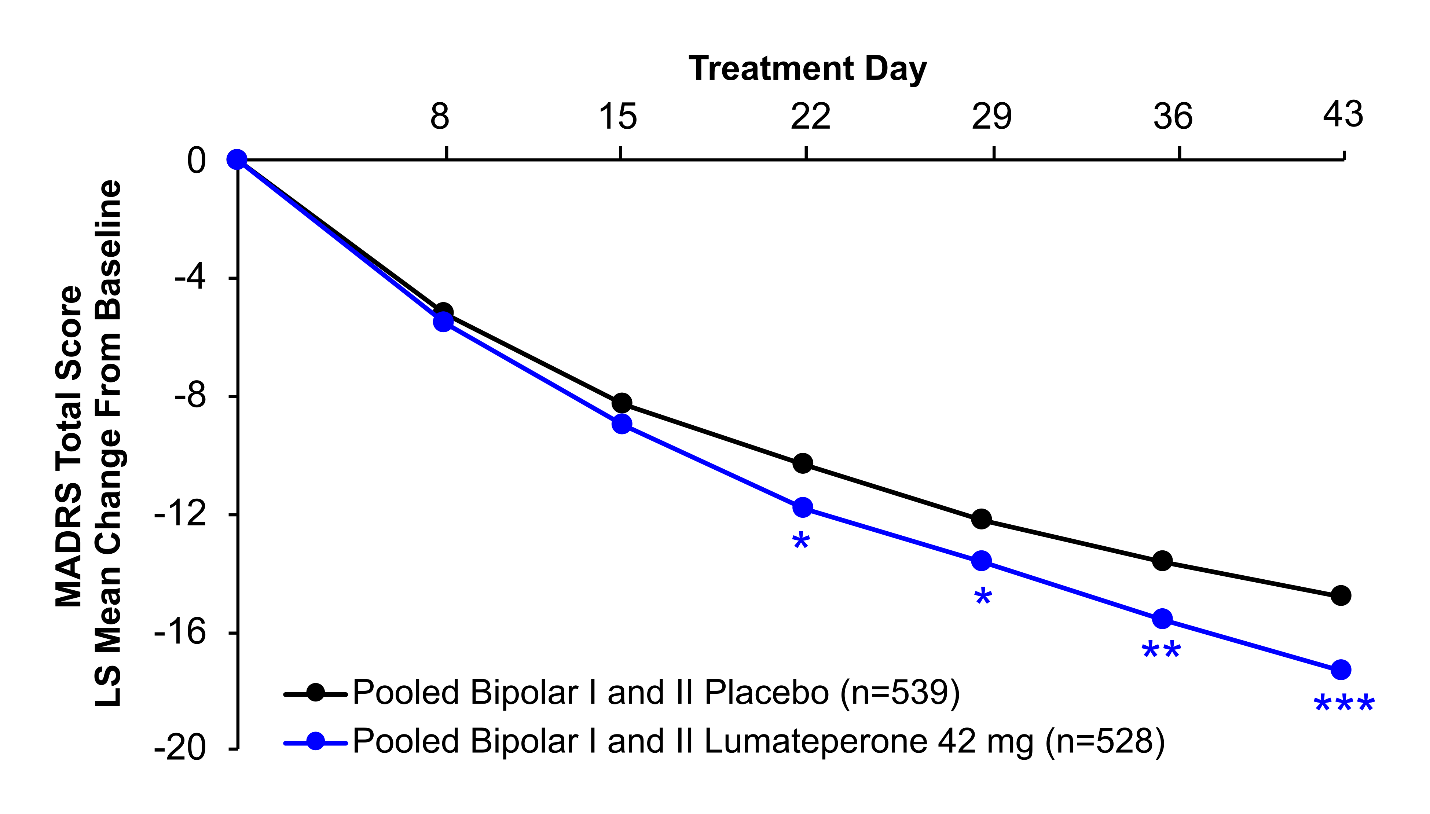
**

**P*<.05 ***P*<.01 ****P*<.001. LSMD vs placebo. MMRM.

ITT, intent-to-treat; LS, least squares; LSMD, least squares mean difference; MADRS, Montgomery-Åsberg Depression Rating Scale; MMRM, mixed-effects model for repeated measures.

# **Supplemental Table S1. Mean Change From Baseline to EOT in Clinician-Rated Motor Scales in Pooled Bipolar II Population (Safety Population)**

| **Scale, LS mean change (SE)** | **Placebo**  **(n=89)** | **Lumateperone 42 mg**  **(n=89)** |
| --- | --- | --- |
| **BARS** | −0.08 (0.04) | −0.05 (0.04) |
| **AIMS** | −0.01 (0.03) | −0.01 (0.03) |
| **SAS** | 0.21 (0.06) | 0.13 (0.06) |

AIMS, Abnormal Involuntary Movement Scale; BARS; Barnes Akathisia Rating Scale; EOT, end of treatment; LS, least squares; SAS, Simpson-Angus Scale.

# **Supplemental Table S2. Change in Body Morphology and Laboratory Parameters at EOT in Pooled Bipolar II Population (Safety Population)**

|  | **Placebo**  **(n=89)** | | **Lumateperone 42 mg**  **(n=89)** | |
| --- | --- | --- | --- | --- |
|  | **Baseline mean (SD)** | **LS mean change (SE)** | **Baseline mean (SD)** | **LS mean change (SE)** |
| **Weight, kg** | 76.31 (14.45) | 0.10 (0.19) | 78.83 (14.31) | 0.25 (0.20) |
|  | **n** | **%^a^** | **n** | **%^a^** |
| ≥7% weight increase | 1 | 1.2 | 0 | 0 |
| ≥7% weight decrease | 1 | 1.2 | 0 | 0 |
|  | **Baseline mean (SD)** | **LS mean change (SE)** | **Baseline mean (SD)** | **LS mean change (SE)** |
| **BMI, kg/m^2^** | 26.11 (3.89) | 0.06 (0.07) | 26.87 (4.32) | 0.07 (0.07) |
| **Waist circumference, cm** | 90.81 (14.17) | −0.10 (0.25) | 91.56 (13.12) | 0.44 (0.26) |
| **Cholesterol, mg/dL** |  |  |  |  |
| Total | 190.54 (39.95) | 0.02 (3.23) | 184.36 (42.94) | 4.42 (3.29) |
| LDL | 110.76 (34.02) | −0.02 (2.75) | 110.23 (36.66) | 2.65 (2.78) |
| HDL | 52.82 (15.34) | −0.15 (0.94) | 50.25 (14.94) | 0.34 (0.96) |
| **Triglycerides, mg/dL** | 140.29 (93.14) | −2.19 (6.01) | 121.91 (68.72) | 2.64 (6.12) |
| **Glucose, mg/dL** | 93.77 (13.01) | −1.19 (1.23) | 94.57 (13.74) | 0.91 (1.26) |
| **Insulin, mIU/L** | 14.58 (13.12) | −0.98 (1.54) | 13.96 (13.46) | 1.09 (1.58) |
| **Prolactin, μg/L** | 13.69 (16.22) | 6.87 (2.48) | 12.17 (10.44) | 0.96 (2.52) |
| **Alanine aminotransferase, U/L** | 20.01 (11.92) | 1.81 (1.38) | 19.36 (11.64) | −0.53 (1.39) |
| **Aspartate aminotransferase, U/L** | 20.48 (8.25) | 0.76 (0.89) | 20.51 (9.23) | −0.06 (0.90) |

^a^Percentage of patients with at least 1 postbaseline value who met the criterion at least once during the double-blind treatment period.

BMI, body mass index; EOT, end of treatment; HDL, high-density lipoprotein; LDL, low-density lipoprotein; LS, least squares.
